# Bacterial safety study of the production process of hemoglobin-based oxygen carriers

**DOI:** 10.3762/bjnano.13.8

**Published:** 2022-01-24

**Authors:** Axel Steffen, Yu Xiong, Radostina Georgieva, Ulrich Kalus, Hans Bäumler

**Affiliations:** 1 Institute of Transfusion Medicine, Charité - Universitätsmedizin Berlin, 10117 Berlin, Germany; 2 Department of Medical Physics, Biophysics and Radiology, Faculty of Medicine, Trakia University, 6000 Stara Zagora, Bulgaria

**Keywords:** bacterial safety, EDTA, glutaraldehyde, hemoglobin, microparticles

## Abstract

Hemoglobin microparticles (HbMP) produced with a three-step procedure, including coprecipitation of hemoglobin with manganese carbonate, protein cross-linking, and dissolution of the carbonate template were shown to be suitable for application as artificial oxygen carriers. First preclinical safety investigations delivered promising results. Bacterial safety plays a decisive role during the production of HbMP. Therefore, the bioburden and endotoxin content of the starting materials (especially hemoglobin) and the final particle suspension are intensively tested. However, some bacteria may not be detected by standard tests due to low concentration. The aim of this study was to investigate how these bacteria would behave in the fabrication process. Biocidal effects are known for glutaraldehyde and for ethylenediaminetetraacetic acid, chemicals that are used in the fabrication process of HbMP. It was shown that both chemicals prevent bacterial growth at the concentrations used during HbMP fabrication. In addition, the particle production was carried out with hemoglobin solutions spiked with *Escherichia coli* or *Staphylococcus epidermidis*. No living bacteria could be detected in the final particle suspensions. Therefore, we conclude that the HbMP fabrication procedure is safe in respect of bacterial contamination.

## Introduction

Artificial oxygen carriers used as red blood cell (RBC) substitutes have attracted particular attention in the last years. Many of the different approaches are using hemoglobin as a starting material to produce hemoglobin-based oxygen carriers (HBOC). Thus, hemoglobin from human and animal (especially bovine) sources or recombinant hemoglobin is used, which can then be chemically modified, cross-linked, polymerized, or encapsulated by various methods [1–3]. We produce biopolymer microparticles as HBOC with the simple coprecipitation–cross-linking–dissolution (CCD) technique while utilizing hemoglobin. Depending on the biopolymer used, there are also various other possible applications for these microparticles. For example, enzyme particles have been produced to be used as microreactors or biosensors [4]. This method can also represent a promising approach to the production of drug carriers by the precipitation of favorable biopolymers and corresponding surface modifications [5–6]. Thus, it was possible to immobilize vitamin B2 (riboflavin) in these particles together with human serum albumin (HSA). This resulted in a drug delivery system with good hemocompatibility and release of riboflavin over a prolonged period [7]. In addition, HSA microparticles could be loaded with doxorubicin, a cytostatic drug used in chemotherapy for cancer treatment. These particles showed higher efficacy in inhibiting metabolic activity in cell culture in comparison to free doxorubicin [8]. To be used as an artificial oxygen carrier, hemoglobin is isolated from bovine blood. Compared to human hemoglobin, it is available in large quantities and free of human pathogens. A comprehensive concept for the biosafety of the bovine hemoglobin as a pharmaceutical starting material has been developed considering the recommendations from the local authorities (Paul-Ehrlich-Institut, Langen, Germany). Among other things, the focus is on the origin and traceability of bovine blood back to the individual animal. It is derived in Tyrol, Austria, an area that is free from bovine spongiform encephalopathy (BSE) according to the World Organization for Animal Health (OIE) [9]. In addition, the animals are declared fit for human consumption after a post-mortem inspection. In the geographical area where the blood is collected, many critical viral pathogens do not occur [10]. Nevertheless, the blood is tested for viral contamination. In terms of bacterial safety, the blood and the hemoglobin obtained from it are tested for bacterial load. In addition, the hemoglobin is tested for endotoxins before its use as a starting material. Only if all specifications are met, the hemoglobin is used for the fabrication of hemoglobin microparticles (HbMP) by the means of the CCD technique [11–12]. During the first step (i.e., coprecipitation) two salt solutions and hemoglobin are mixed. A salt template is created in which hemoglobin is trapped. In the next main step (i.e., cross-linking) the individual hemoglobin molecules are polymerized in the salt template. The third step is dissolution. Dissolving of the salt template by adding ethylenediaminetetraacetic acid (EDTA) results in submicrometer hemoglobin particles. The resulting particles have an advantageous oxygen affinity and show a narrow size distribution of approx. 700 nm. In the first pre-clinical investigations it was shown that HbMP meet the requirements as a novel artificial oxygen carrier for application as a blood substitute and are considered nonmutagenic by different in vitro and in vivo studies [13].

Although all substances used to produce HbMP are either of pharmaceutical grade or approved drugs (HSA), except for hemoglobin, the sterility of the final product is not guaranteed. Terminal sterilization of hemoglobin as well as particle suspension with standard methods of heat inactivation, UV-C irradiation, or gamma irradiation all led to a denaturation of the hemoglobin or to an enormous formation of methemoglobin due to the oxidation of iron in the heme group. Methemoglobin is not able to release oxygen [14–15]. It is, therefore, not suitable for the use in the production of HbMP applied as an artificial oxygen carrier. Since the aforementioned sterilization methods cannot be used, the solution to obtain sterile hemoglobin is to sterile filter it after production and test it for endotoxin content and bioburden. This way, bacterial contamination is relatively unlikely. Nevertheless, it is conceivable that a minimal amount of bacteria will not be detected by the tests. In this case, it would be advantageous if any additional bacterial depletion could be achieved by steps in the production process. In comparison with the manufacturing of human blood products, it should be noted that the starting materials can be tested or are declared by the manufacturer to be sterile or endotoxin-free. The subsequent manufacturing process for human blood products is so safe that random testing of the products is sufficient. By adopting this principle, each batch is be tested, but not every single product.

Endotoxins or the lipopolysaccharide (LPS) of the outer membrane of Gram-negative bacteria is another important point regarding safety. Endotoxins could potentially be introduced into the production process by the starting substances. Also, the depletion of any bacteria potentially present in the process could also cause the LPS to be released. One *Escherichia coli* cell has approximately 10–50 fg of LPS [16–17]. One endotoxin unit (EU) corresponds to 100 pg of *E.coli* LPS or a bacterial count in the range of 10^4^ cells/mL [18–19]. According to the US and European Pharmacopoeia, the endotoxin limit for intravenous administration of a drug is 5 EU/kg. Taking an example of a hypothetical human body of 70 kg, this leads to a dose of 350 EU per administration. If one wants to administer a quantity of 250 mL of HbMP, the suspension must not contain more than 1.4 EU/mL, for an administration of 500 mL, which corresponds to 0.7 EU/mL. Our limit for endotoxin load is 0.5 EU/mL and this limit is tested before the release of the product.

The production process includes several washing steps that could contribute to the depletion of a potential bacterial load. Glutaraldehyde (GA) is used for the inter- and, to a certain extent, intramolecular cross-linking of the hemoglobin molecules at a concentration of 0.02% [20–21]. It is known to have an antibacterial effect and it is used as a disinfectant or for cold sterilization of medical instruments in hospitals at higher concentrations [22–24]. Glutaraldehyde is also widely used in biochemical applications and as a fixative for electron microscopy [25–26].

EDTA is used in the HbMP fabrication process to dissolve the manganese carbonate template to produce pure protein particles. EDTA is widely utilized in medical and biological applications. Because of its chelating properties, it is used as an anticoagulant in blood samples [27]. It has also long been used to permeabilize the cell wall of Gram-negative cells [28–29]. A certain inhibitory effect of EDTA on the growth of *Staphylococcus epidermidis* could also be shown [30].

Therefore, the aim of this work was to investigate whether the steps of cross-linking with GA or dissolution with EDTA, in addition to the washing steps in the particle production process, can contribute to the reduction of a potential contamination with Gram-positive and Gram-negative bacteria. *Staphylococcus epidermidis* and *Escherichia coli* were selected as model organisms for this purpose. Both bacteria have been intensively studied. *E. coli* is mainly found in the intestines of humans and animals, is Gram-negative, and has an approximate length of 2 µm with a diameter of 1 µm and a cylindrical shape [31]. *S. epidermidis* lives on human skin but is also frequently responsible for infections of immunocompromised patients in hospitals [32–33]. This Gram-positive bacterium has a spherical shape and a diameter of 0.5–1.5 µm [34]. In addition, both bacteria play a role in adverse transfusion reactions [35]. Due to storage conditions at room temperature, mainly platelet concentrates are affected [36–37]. Here, contamination with *S. epidermidis* occurs in particular due to the colonization of the skin and inadequate disinfection of the puncture site during blood donation [38–39]. As a result, bacterial contamination that is not detected by testing could occur during the preparation of blood products as well as during the production of HbMP.

To investigate the possible inhibitory effects of the chemicals used in the CCD process, we assessed the growth of bacteria upon the addition of GA and EDTA to the growth medium. In addition, HbMP fabricated with bacteria-spiked hemoglobin were produced and the bacterial load was examined at every step of the particle production process.

## Results and Discussion

Various tests were carried out to find out whether any possible bacterial contamination was removed during the HbMP production process. For this purpose, the influence of the chemicals glutaraldehyde and EDTA on Gram-negative and Gram-positive bacteria was investigated in preliminary experiments. Glutaraldehyde is used in the HbMP manufacturing process to cross-link proteins. EDTA is used to dissolve the carbonate template.

In order to check whether glutaraldehyde and EDTA have an influence on the bacterial safety of HbMP, growth tests were first carried out with Gram-positive and Gram-negative bacterial cultures in the presence of these substances. The model organism *Escherichia coli* was chosen as a representative of Gram-negative bacteria, and *Staphylococcus epidermidis* served as an example of a Gram-positive bacterium.

Additionally, HbMP were prepared with a spiked hemoglobin solution as well as under standard process conditions as a control.

### Hemoglobin microparticles – size, zeta potential, morphology

**Figure 1:**
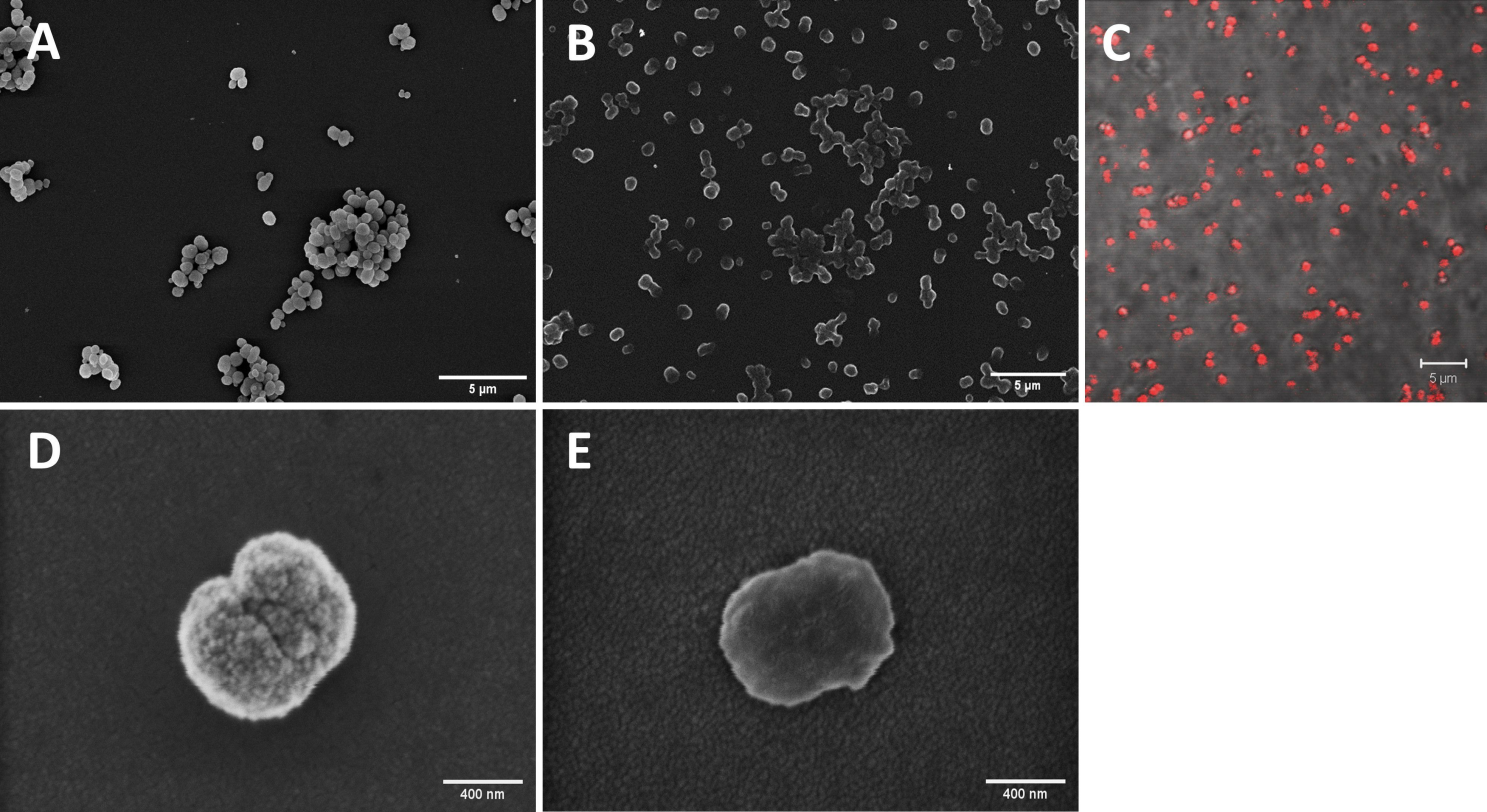
Microscopic images of HbMP. (A, D) SEM images of dried and adherent particles after precipitation with carbonate template. (B, E) SEM images of dried and adherent final HbMP after precipitation, cross-linking, dissolution, and washing. (C) CLSM image with autofluorescent HbMP in suspension. Scale bars: A, B, C = 5 µm, D, E = 400 nm.

In addition to particle preparation with spiked hemoglobin, particles were also prepared using the standard protocol. The CCD method produces nearly uniform, peanut-shaped particles. The size distribution determined by dynamic light scattering (DLS) was 759 ± 25 nm. Confocal laser scanning microscopy (CLSM) images confirmed this size range (Figure 1C). Scanning electron microscopy (SEM) images of particles produced with the CCD method after precipitation, as well as after cross-linking, dissolution, and final washing steps are shown in Figure 1.

The zeta potential of HbMP in phosphate-buffered saline (PBS), pH 7.4, was −8.51 ± 0.9 mV. The zeta potential in PBS, pH 7.0, of *E. coli* was −16 mV, that of *S. epidermidis*, −8 mV [40]. Thus, both the HbMP and the bacteria showed a negative zeta potential and strong aggregation due to different charges seems unlikely. In the CCD process there are some centrifugation steps in the preparation of the HbMP. After centrifugation, both the particles and the majority of the bacteria were found in the fraction of the sediment.

### Influence of glutaraldehyde on bacterial growth

**Figure 2:**
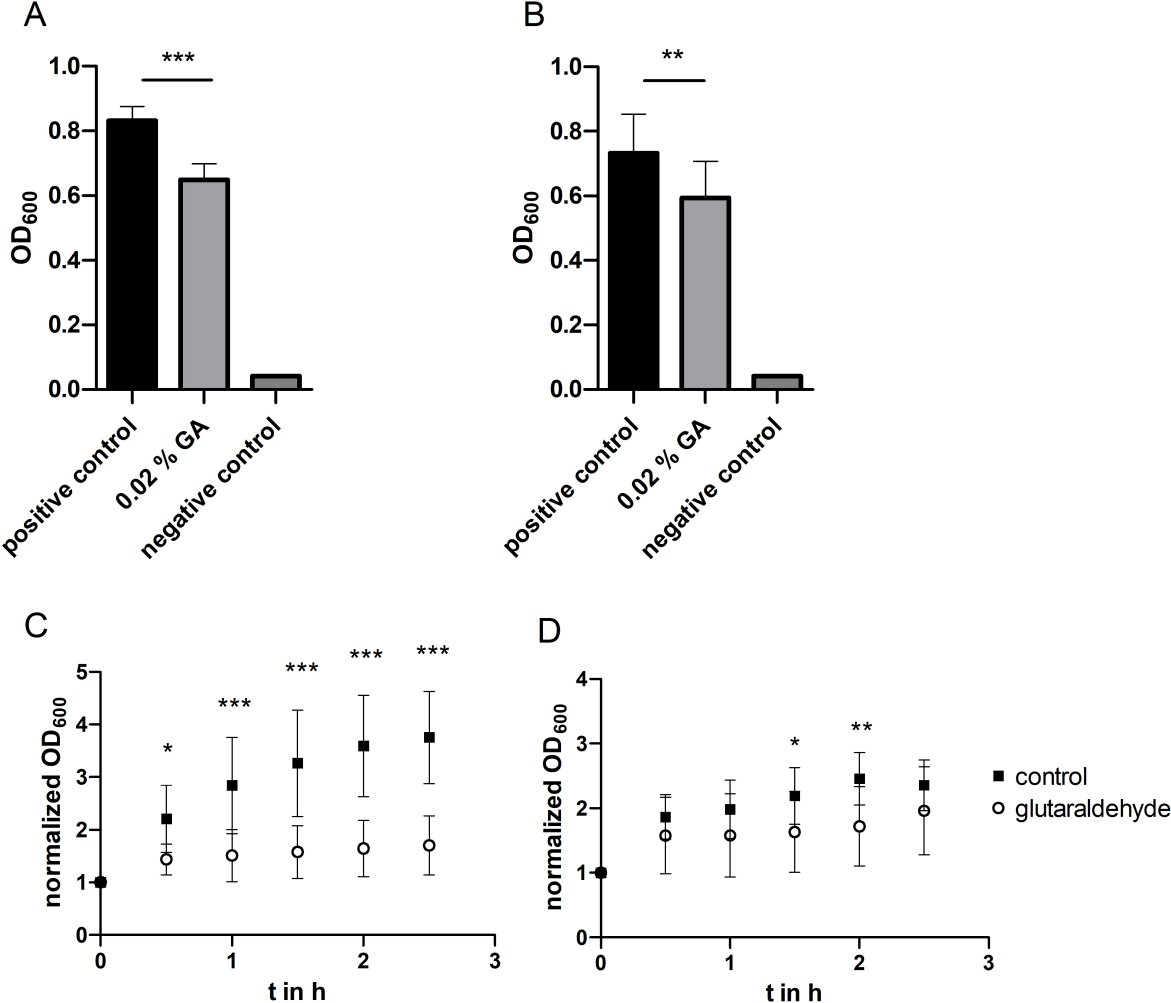
Effect of glutaraldehyde on bacterial growth of *E. coli* and *S. epidermidis*. *E. coli* (A) and *S. epidermidis* (B) cells were incubated for 20 h at 37 °C in Mueller–Hinton II Bouillon (positive control), Mueller–Hinton II Bouillon + 0.02% GA, or Mueller–Hinton II Bouillon + 0.4% peracetic acid (negative control). Error bars represent standard deviation (SD, *n* = 12 biological replicates). The statistical analysis was performed by one-way ANOVA with Bonferroni’s multiple comparison test, SD for negative controls <0.0015. *E. coli* (C) and *S. epidermidis* (D) cells were preincubated for 20 h at 37 °C in Mueller–Hinton II Bouillon. Afterwards the growth medium was removed and replaced with fresh Mueller–Hinton II Bouillon (“control”) or Mueller–Hinton II Bouillon + 0.02% GA (“glutaraldehyde”). Cells were grown for 2.5 h at room temperature. Error bars are SD (*n* = 12 biological replicates). Statistical analysis was performed by a two-way ANOVA with Bonferroni’s multiple comparison test; * corresponds to *p* < 0.05; ** corresponds to *p* < 0.01; *** corresponds to *p* < 0.001.

*E. coli* cells cultivated with 0.02% glutaraldehyde at 37 °C showed a significantly reduced growth compared to the control group in normal growth medium (Figure 2A). However, the growth of bacteria was stronger than that in the negative control with peracetic acid. This means that the multiplication of bacteria still occurred to some degree. Glutaraldehyde inhibits viable functions so that the bacteria are subsequently unable to proliferate, but it also fixes the cell wall components. Thus, the cells are not lysed. The presence of bacteria was detected in the experiments by determining the optical density. The fixed but dead cells thus explain the higher signal compared to the negative control. The cultivation of *S. epidermidis* with glutaraldehyde at 37 °C delivered similar results (Figure 2B). Glutaraldehyde significantly inhibits the proliferation of bacteria under the given conditions.

During cultivation of *E. coli* with the addition of glutaraldehyde at room temperature, there were also significant differences in growth rates compared to the control (Figure 2C). After a small increase in the optical density after the measurement point at 30 min, there was no further growth of the cells. In contrast, the cells in the control group (in normal growth medium) continued to strongly grow over the course of the experiment. For better comparability, the optical density values here were normalized to the respective initial value. A similar, albeit not as pronounced, scenario emerged when *S. epidermidis* was cultivated at room temperature (Figure 2D). Here too, the optical density increased after the measuring point at 30 min, almost to the range of the control. However, it subsequently remained at this level while the control cells continued to grow. The smaller difference between the control group and cells cultured with glutaraldehyde compared to the *E. coli* growth curves could also be due to the longer generation time of *S. epidermidis*.

**Figure 3:**
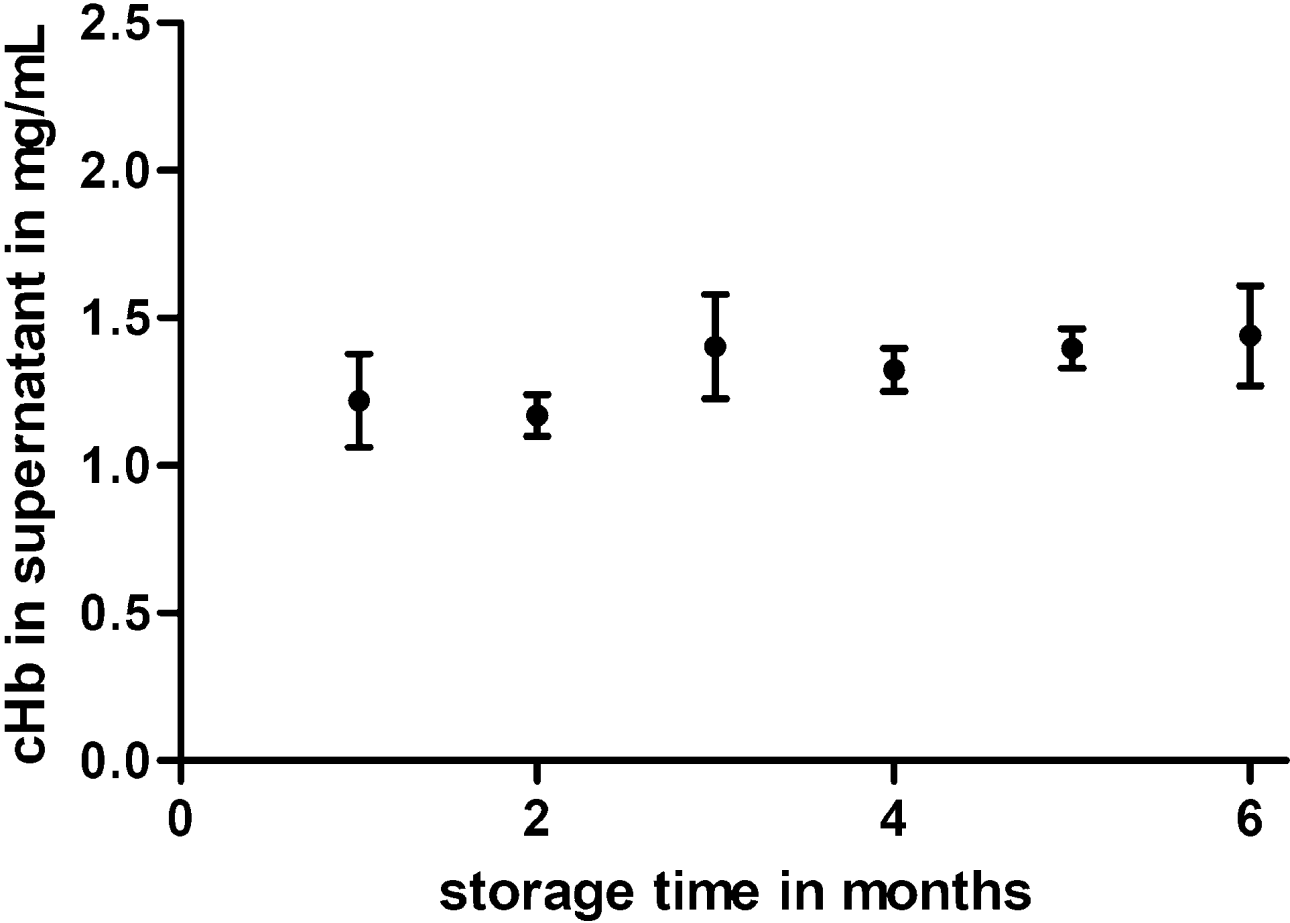
Concentration of free hemoglobin in the supernatant of HbMP suspensions. Measuring points are means with SD from three batches of HbMP.

Glutaraldehyde in higher concentrations is widely used as a biocide. It is mainly applied to disinfect surfaces or medical instruments [41–43]. Glutaraldehyde is applied in the manufacturing process of HbMP to cross-link hemoglobin molecules. This cross-linking is also the main reason for the biocidal effect. Glutaraldehyde strongly reacts with proteins and can inhibit DNA synthesis in bacteria, and similar effects are also seen on RNA and protein syntheses [44–45]. In addition, glutaraldehyde particularly acts on the outer layers of *E. coli* and cross-links lipoproteins and proteins there as well. This fixation of bacteria prevents the bacterial cells from multiplying. Permeabilization of the cell wall and leakage of intracellular material thereby do not take place [46]. Similar effects have also been shown for *S. epidermidis*. Glutaraldehyde can also kill the bacteria in this case but does not permeabilize the cell wall [47–48]. In the manufacturing process of HbMP, a GA concentration of 0.02% was used. A study of stability testing was carried out to confirm that this GA concentration was sufficient for the production of HbMP. Three batches of HbMP (20% (v/v) particle concentration, hemoglobin content: 24.5 ± 1.2 mg/mL) were aliquoted (10 mL) and stored at 2–8 °C. Every month an aliquot per batch was analyzed for the amount of released hemoglobin. As shown in Figure 3, the concentration of free hemoglobin remained almost constant over the measurement period in the range of 1 to 1.5 mg/mL and, thus, in a similar range as the amount of free hemoglobin allowed for erythrocyte concentrates during their storage period [49–50]. No additional release of hemoglobin was observed; therefore, the particles prepared with 0.02% GA are stable for at least six months. A higher concentration of GA was not used in the particle fabrication process since it caused a higher phagocytosis rate of HbMP and led to an increased amount of methemoglobin [51].

### Effect of EDTA on bacterial growth

**Figure 4:**
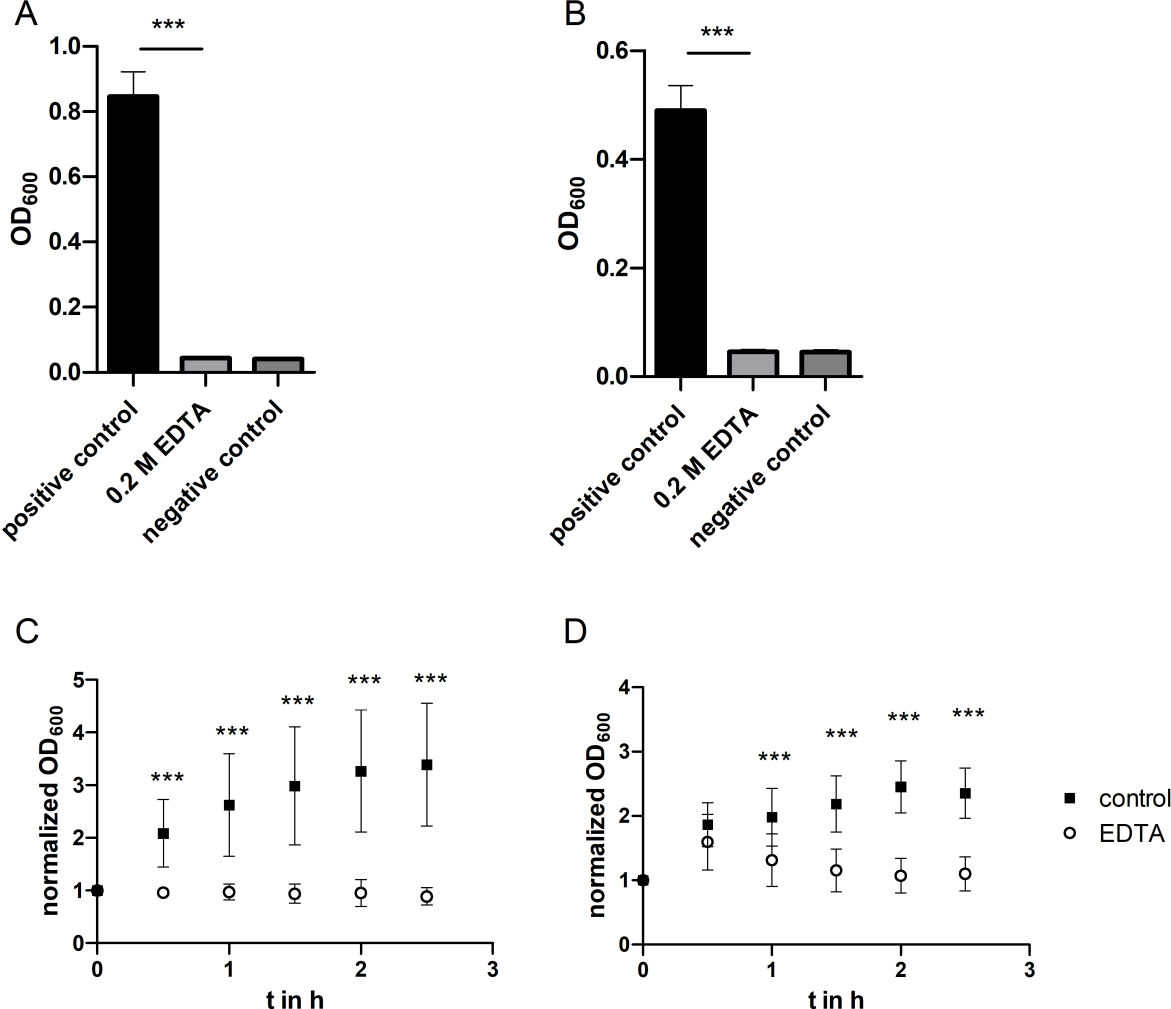
Effect of EDTA on bacterial growth of *E. coli* and *S. epidermidis*. *E. coli* (A) and *S. epidermidis* (B) cells were incubated for 20 h at 37 °C in Mueller–Hinton II Bouillon (positive control), Mueller–Hinton II Bouillon + 0.2 M EDTA, or Mueller–Hinton II Bouillon + 0.4% peracetic acid (negative control). Error bars are SD (*n* = 12 biological replicates). SD for negative controls and 0.2 M EDTA <0.005. *E. coli* (C) and *S. epidermidis* (D) cells were preincubated for 20 h at 37 °C in Mueller–Hinton II Bouillon. Afterwards, the growth medium was removed and replaced with fresh Mueller–Hinton II Bouillon (“control”) or Mueller–Hinton II Bouillon + 0.2 M EDTA (“EDTA”). Cells were grown for 2.5 h at room temperature. Error bars are SD (*n* = 12 biological replicates). Statistical analysis was performed by a two-way ANOVA Bonferroni’s multiple comparison test; *** corresponds to *p* < 0.001.

*E. coli* (Figure 4A) and *S. epidermidis* (Figure 4B) were cultivated in growth medium containing 0.2 M of EDTA at 37 °C. In contrast to the experiments in which glutaraldehyde was added, here both bacterial strains showed no growth compared to controls in media without additives. The bacterial growth, as determined by the optical density of the sample, was equivalent to that of the negative control in peracetic acid. The addition of EDTA to the growth medium led to a complete inhibition of bacterial proliferation.

There was also no growth during incubation of *E. coli* upon addition of EDTA at room temperature (Figure 4C). The optical density did not increase at any time point compared to the initial value. In contrast, the bacteria in the control group, in normal medium, grew strongly. Again, optical density values were normalized to the respective initial value for better comparability. The *S. epidermidis* cells of the control group showed a similar growth behavior as in the experimental group with glutaraldehyde addition (Figure 4D). The cells incubated with EDTA showed some increase in optical density at the measurement point after 30 min. However, this drops back to the initial value in the further course of the experiment. Thus, there is no growth of either bacterial strains in this experimental arrangement.

EDTA as a chelating agent is known to bind divalent cations. Especially the chelation of Mg^2+^ results in a destabilization of the negative charges of the outer membrane of Gram-negative bacteria [45]. The cells thus become permeable. This effect can be exploited to make the cells more receptive to antibiotics, biocides, or other substances [28,52]. Depending on the concentration of EDTA and the bacterial strain, there is a release of membrane components, proteins, and finally cell lysis [53–55]. The treatment of Gram-negative cells with EDTA can lead to a release of up to 50% of the LPS from the cell wall of a bacterium [56–57]. These effects have been demonstrated for Gram-negative bacteria, especially *E. coli* [29,58]. However, EDTA can also lead to inhibition of growth and cell lysis in Gram-positive bacteria [59–60].

In the study presented here, EDTA is used to dissolve the carbonate template during particle preparation at a concentration of 0.2 M. The results of our experiments (Figure 4) are in agreement with the abovementioned literature.

### Combination of glutaraldehyde and EDTA according to the particle preparation routine

**Figure 5:**
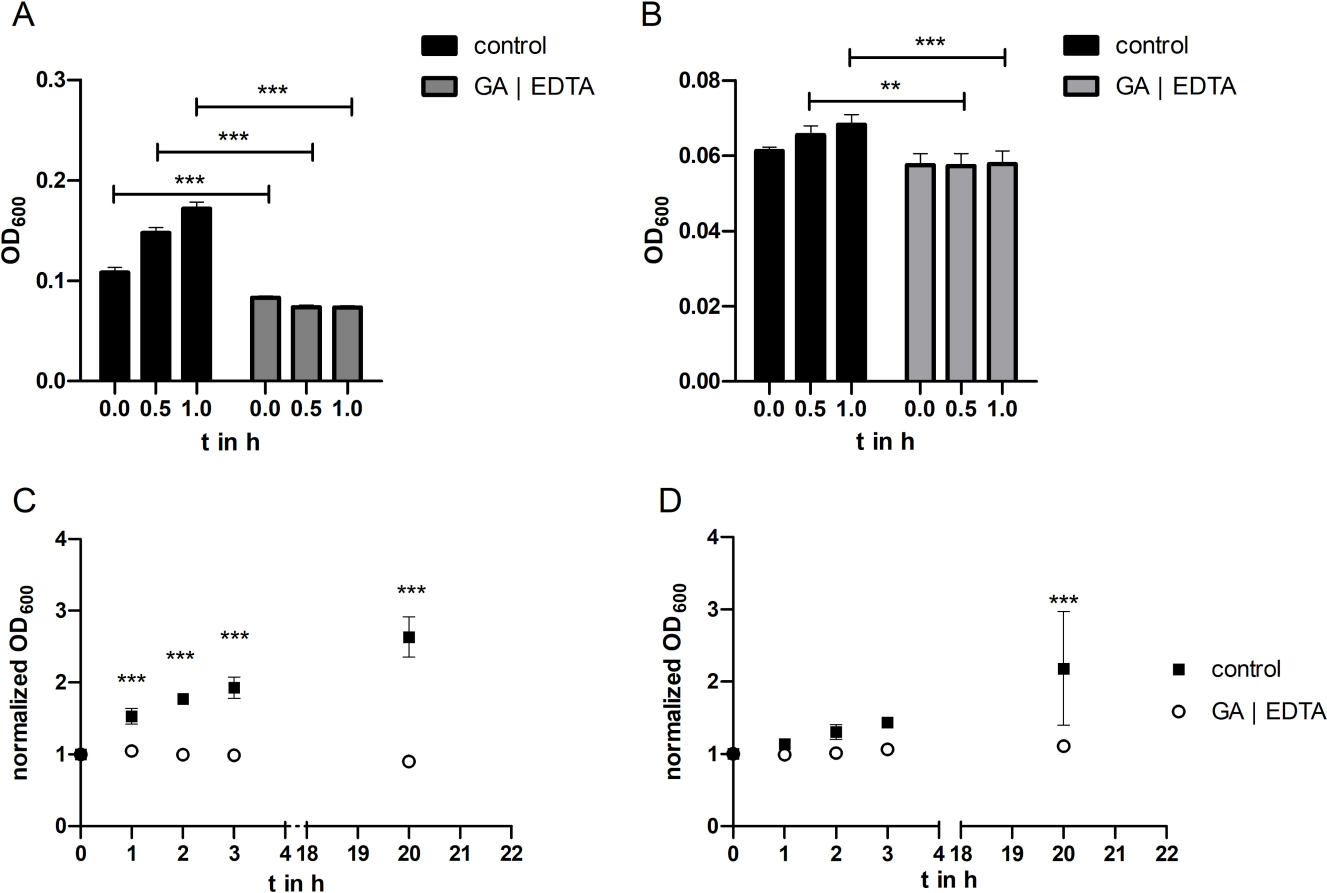
Effect of a combination of glutaraldehyde and EDTA on *E. coli* and *S. epidermidis*. *E. coli* (A) and *S. epidermidis* (B) cells were grown in Mueller–Hinton II Bouillon (“control”) and Mueller–Hinton II Bouillon supplemented with 0.02% glutaraldehyde and 0.2 M EDTA (“GA | EDTA”) for one hour at room temperature. SD for GA | EDTA in (A) is 0.005. (C, D) Cells grown as described in (A) and (B) were subsequently incubated at 37 °C for 20 h. Error bars are SD (*n* = 4 biological replicates). Statistical analysis was performed by a two-way ANOVA with Bonferroni’s multiple comparison test; ** corresponds to *p* < 0.01; *** corresponds to *p* < 0.001.

In addition to the experiments described above, in which the effect of glutaraldehyde and EDTA was separately examined, both substances were combined with one another in further experiments. It should be investigated whether the effect of the two substances can cancel or strengthen each other. The concentrations of GA and EDTA corresponded to those used in the fabrication process. The results are shown in Figure 5.

When *E. coli* bacteria were cultured with the addition of EDTA and GA at room temperature for 1 h, there was no increase in optical density and thus no growth of bacteria (Figure 5A). In contrast, control cells grew normally. In the corresponding growth experiments with *S. epidermidis*, there was also no increase in optical density after addition of EDTA and GA (Figure 5B). The cells of the control group did not grow as much here, but the differences in growth were significant during the experiment compared to the cells cultured with additives.

There was also no detectable growth of *E. coli* after the addition of EDTA and GA and cultivation at 37 °C (Figure 5C). The optical density remained almost constant during the experiment compared to the initial value. The control cells grew strongly, so there were significant differences in growth at each measurement time point in this experiment. When culturing *S. epidermidis* after the addition of EDTA and GA, no growth was seen either. However, again, the control cells did not grow as much. Nevertheless, at the end of the experiment, the difference in growth compared to the control was significant (Figure 5D).

These experiments also confirm the growth-inhibiting effect of EDTA and GA, also in combination, in the concentrations used in the HbMP manufacturing process routine.

### Particle preparation with the addition of bacteria

**Figure 6:**
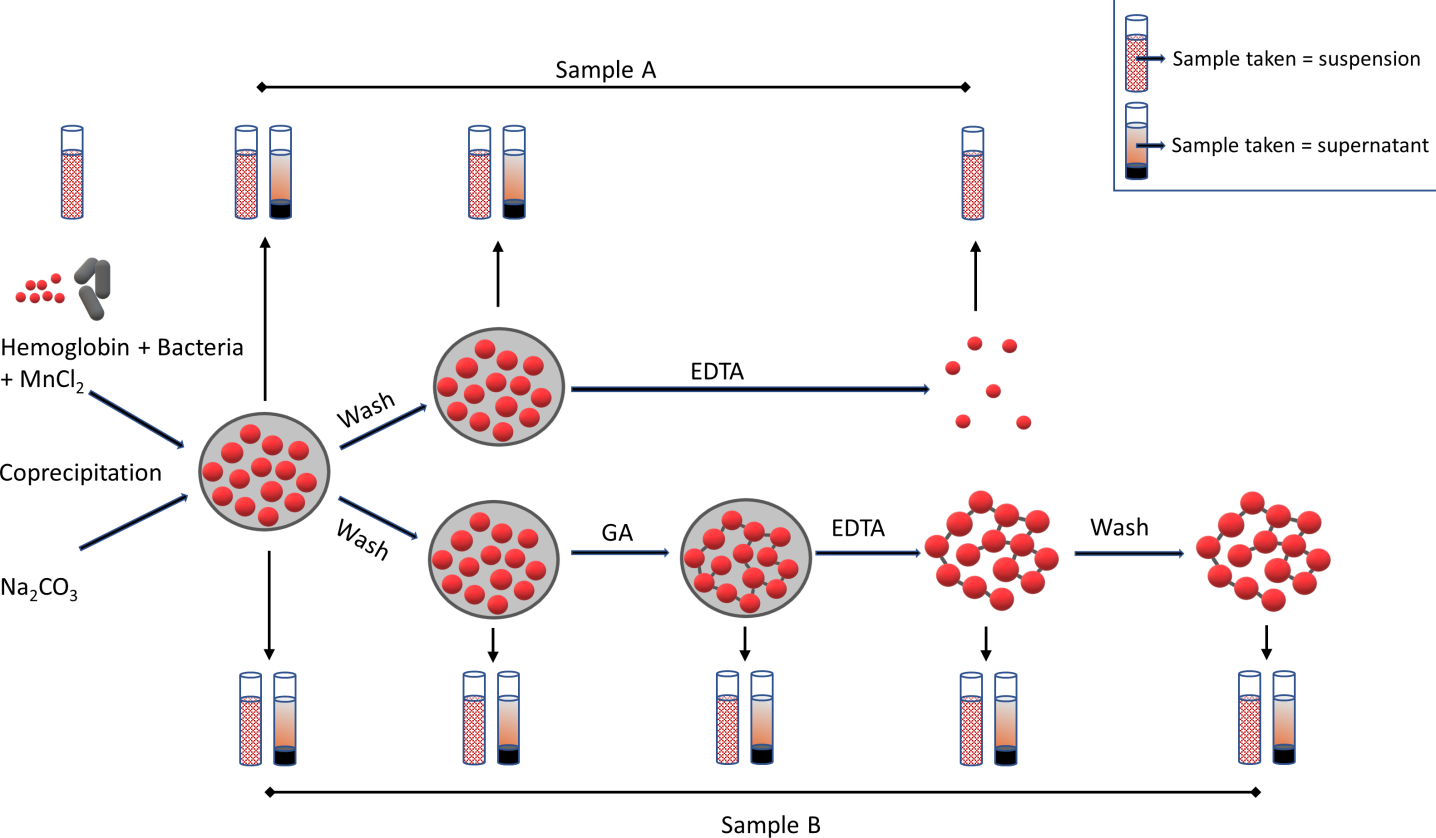
Simplified scheme of the experimental approach. The solution containing hemoglobin and MnCl_2_ was spiked with *E. coli* or *S. epidermidis*, respectively, and coprecipitated with Na_2_CO_3_. The resulting particles were washed with NaCl and either the salt template was directly dissolved with EDTA (sample A) or the particles were cross-linked with glutaraldehyde (GA) and then dissolved with EDTA (sample B). At various production steps, the number of colony-forming units (CFU) was determined in the complete sample (suspension) and in the supernatant after centrifugation, respectively.

The experiments described above have shown the inhibitory effect of glutaraldehyde and EDTA on the growth of *E. coli* and *S. epidermidis* when the substances were added to the growth medium. Regarding the production of HbMP, however, it is of particular interest whether bacterial contamination can also be removed during the production process. To investigate the growth of both bacteria during the HbMP production process, the initial hemoglobin solution was spiked with *E. coli* and *S. epidermidis*, respectively, and particle production was performed (Figure 6).

**Figure 7:**
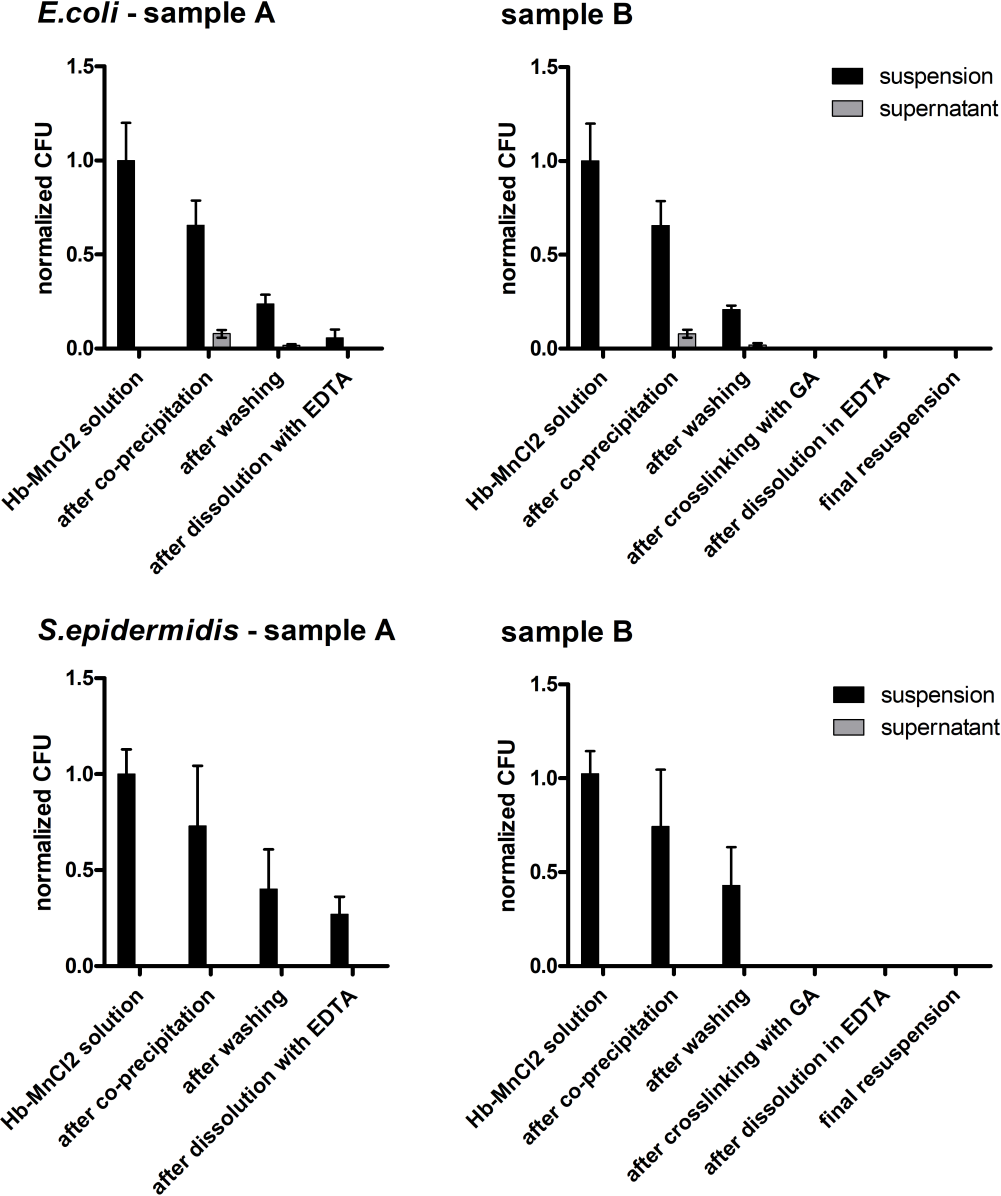
Particle preparation with *E. coli* and *S. epidermidis*. The solution containing hemoglobin and MnCl_2_ was spiked with *E. coli* (upper row) or *S. epidermidis* (lower row) and coprecipitated with Na_2_CO_3_. The resulting particles were washed with NaCl and either the salt template was directly dissolved (sample A) or the particles were cross-linked with glutaraldehyde and then dissolved with EDTA (sample B). At each given production step, the number of colony-forming units was determined. Error bars are SD, *n* = 8, except for the “Hb–MnCl_2_ solution” group (*n* = 4).

The samples were taken after each production step and checked to see if viable bacteria were still present. The samples were centrifuged and the amount of bacteria in the total sample suspension (before centrifugation) as well as in the supernatant (after centrifugation) was examined (Figure 7).

After precipitation, most of the original bacterial count of both bacterial strains was still present in the suspension. In the experiment with *E. coli*, a small proportion was detectable in the supernatant of the sample (Figure 7, upper row, sample A). This means that a large part of the bacteria was in the sediment (i.e., in the particle fraction). After the first washing step in the process, the detectable bacterial count was further reduced to about one quarter of the initial value. In this series of experiments, the proteins in the particles were not cross-linked, but the carbonate templates were immediately dissolved with EDTA resulting in no particle formation (see Figure 6 – sample A). After resuspension of the particles in EDTA, only a few of the originally used bacteria were still present. The results were similar for particle preparation with hemoglobin spiked with *S. epidermidis* (Figure 7, lower row, sample A). However, after the final dissolution step with EDTA, the sample still contained about a quarter of the initial number of viable bacteria. In another series of experiments, the particles were cross-linked with glutaraldehyde before dissolution with EDTA. This corresponded to the actual HbMP production process (Figure 6 – sample B). Here it could be seen that after cross-linking with glutaraldehyde, no viable bacteria of either strain were detectable (Figure 7 – samples B).

In these experiments, the above observations could be confirmed. The concentrations of GA and EDTA used in the CCD process were sufficient to cross-link the hemoglobin molecules and dissolve the carbonate template. Furthermore, both chemicals resulted in no detectable viable bacteria at the end of the particle production process. In the above experiment, the EDTA concentration optimized for the CCD process alone was not sufficient to remove all bacteria from the solution without cross-linking the particles with GA. EDTA forms chelate complexes with metal ions, which are thereby incorporated into a ring structure. The formed complexes with manganese ions are stronger than the complexes formed with magnesium [61]. For this experiment, it should be noted that EDTA complexes both the manganese from the manganese carbonate template and magnesium ions from the cell wall of the bacteria. The former leads to dissolution of the carbonate template of the particles, while the latter leads to prevention of bacterial proliferation. It is possible that the stronger binding of EDTA to manganese results in insufficient EDTA to bind to all the magnesium from the cell walls of the bacteria. However, if the actual production process of the HbMP was performed, (i.e., including the cross-linking with glutaraldehyde) no more bacteria would be detectable after the cross-linking step.

Thus, the use of GA and EDTA together with washing effects may be part of a comprehensive biological safety concept for the production of HbMP for the potential use as an artificial oxygen carrier and blood substitute.

## Conclusion

In conclusion, we could show that HbMP can be safely produced with respect to bacterial contamination. Biopolymer particles can be produced with the simple CCD technique and promise a wide range of biomedical applications, depending on the biopolymer used. The application of HbMP as artificial oxygen carriers came into focus. Initial preclinical studies yielded promising results. In these particles (i.e., HbMP), hemoglobin is used for particle production and EDTA and glutaraldehyde are applied in the standard production process. The effect of these chemicals in the concentration range used, together with washing effects during production, ensure that any previously undetected bacterial contamination is removed. After production and final determination of the bioburden and endotoxin content (and of course if all other quality control parameters are met) the produced batch of HbMP can be released for further use. These findings are an important part of our extensive safety concept.

## Experimental

### Materials

Ringer's acetate solution was purchased from Serumwerk Bernburg AG, Bernburg, Germany; aqua ad iniectabilia (Ampuwa) from Fresenius Kabi Deutschland GmbH, Bad Homburg, Germany, and sodium chloride (NaCl) from B. Braun Melsungen AG, Melsungen, Germany. Human serum albumin, 200 g/L, Baxalta was obtained from Takeda Pharma Vertrieb GmbH & Co. KG, Berlin, Germany. EDTA, PBS, and glycine were obtained from AppliChem GmbH, Darmstadt, Germany. Manganese dichloride (MnCl_2_), sodium carbonate (Na_2_CO_3_), glutaraldehyde, sodium borohydride (NaBH_4_), sodium hydroxide (NaOH), and Mueller–Hinton II Bouillon were purchased from Merck KGaA, Darmstadt, Germany.

*Staphylococcus epidermidis* (own cultivation) and *Escherichia coli* (ATCC 25922) were kindly provided by the Bereswill Lab, Institute of Microbiology, Infectious Diseases and Immunology, Charité – Universitätsmedizin Berlin, Berlin, Germany. Columbia agar with 5% of sheep blood was purchased from Thermo Fisher Diagnostics GmbH Microbiology, Wesel, Germany.

Hemoglobin was prepared by hypotonic hemolysis as described earlier [62–63] with slight adjustments. Briefly, fresh bovine whole blood (anticoagulated with 2 g/L of EDTA, provided by Biophyll GmbH, Dietersburg, Germany) was centrifuged for 20 min at 2500*g* at 2–8 °C. The resulting packed red blood cells (RBC) were washed at least three times with sterile 0.9% NaCl and lysed overnight with four to five volumes of a low osmotic sodium chloride solution (100 mOsmol/kg). The lysate was centrifuged at 5500*g* at 2–8 °C for 4 h and the supernatant was processed by means of tangential flow filtration (TFF). A KrosFlo KR2 system with a 500 kDa mPES hollow fiber module (Repligen Europe B.V., Breda, Netherlands) was used similarly to the process described earlier [64]. The processed Hb was stored at −80 °C until use.

### Preparation and characterization of hemoglobin microparticles

Hemoglobin microparticles (HbMP) were fabricated by the CCD technique [51,65]. Shortly, 0.25 M of Na_2_CO_3_ and 0.25 M of MnCl_2_ including 10 mg/mL of Hb and 1 mg/mL of HSA were rapidly mixed at room temperature (coprecipitation). After coprecipitation, 2.5 mg/mL of HSA was added and after 5 min the particles were separated by centrifugation and washed three times with 0.9% NaCl. The particles were resuspended in a 0.02% GA solution and incubated for 1 h at room temperature on a shaker (cross-linking). After another centrifugation, the excess GA was quenched with 0.1 M of glycine. A solution of 0.2 M of EDTA pH 7.4 was added to dissolve the MnCO_3_ template (dissolution) and the resulting protein particles were treated with NaBH_4_ in 0.1 M of NaOH. Lastly, the particles were washed three times with 0.9% NaCl and resuspended in Ringer's acetate until further use.

### Characterization of hemoglobin microparticles

For the SEM analysis, one drop of the sample was applied to a glass slide, dried overnight, and sputtered with gold. A Gemini Leo 1550 (Carl Zeiss AG, Oberkochen, Germany) instrument was utilized for the measurements at an operation voltage of 10 kV.

The particle size was measured by DLS applying a Zetasizer Nano ZS instrument (Malvern Panalytical Ltd., Malvern, U.K.). Additionally, CLSM images were taken with a LSM 510 Meta (Carl Zeiss AG, Oberkochen, Germany) confocal microscope and the size was measured from the images. The microscope was used with a 100× oil-immersion objective (numerical aperture 1.3) while utilizing an excitation wavelength of 488 nm and a 505 nm long-pass emission filter.

The zeta potential of HbMP in 0.9% NaCl (pH 7.4, conductivity 17.2 ± 0.9 mS/cm) was measured using the Zetasizer Nano ZS instrument.

For the determination of the concentration of free hemoglobin in the HbMP suspension, aliquots of three batches of HbMP, produced with 0.02% GA were stored at 2–8 °C for up to six months. Every month an aliquot was taken and centrifuged at 20 000*g* for 30 min (Hettich Mikro 22R, Hettich GmbH & Co. KG, Tuttlingen, Germany). The hemoglobin released in the supernatant was measured with a standard alkaline haematin detergent (AHD) [66].

### Preparation of hemoglobin microparticles spiked with bacteria

Hemoglobin microparticles spiked with bacteria were produced by adding *E. coli* or *S. epidermidis*, respectively, to the hemoglobin solution (see Figure 6). The bacteria were spread on agar plates three days prior to particle preparation and incubated at 37 °C. Then, about one third of a bacterial colony was transferred into sterile injection water (Ampuwa), the optical density at 600 nm (OD_600_) was measured (Spectra Classic, Tecan Group Ltd., Maennedorf, Switzerland) and adjusted to 1.5 × 10^8^ CFU/mL. The bacterial suspension was diluted to 2.5 × 10^5^ CFU/mL and mixed with the hemoglobin solution. The concentration of bacteria in the hemoglobin solution was determined for every particle batch and used as an initial value to evaluate bioburden at different steps in the particle preparation process.

### Determination of bacterial growth

#### Influence of glutaraldehyde and EDTA

The bacteria plus EDTA (0.2 M) or GA (0.02%) were filled in the wells of a microtiter plate. The samples contained bacteria at a concentration of approximately 7.5 × 10^5^ CFU/mL. The positive control consisted of bacteria and growth medium (Mueller–Hinton II Bouillon), the negative control of bacteria and 0.4% peracetic acid, and the sterile control consisted of medium only. The bacteria were preincubated for 20 h at 37 °C in Mueller–Hinton II Bouillon. Afterwards, the growth medium was removed and replaced with fresh Mueller–Hinton II Bouillon (control), Mueller–Hinton II Bouillon + 0.02% GA, or 0.2 M of EDTA. Cells were grown for 2.5 h at room temperature. At the start and at different time points, the optical density at 600 nm was determined to assess bacterial growth. In a further experiment, a mixture of EDTA and GA was simultaneously examined.

#### Determination of bioburden during preparation of hemoglobin microparticles

To assess the amount of viable bacteria, samples were taken from the initial Hb–MnCl_2_ solution. After coprecipitation, washing, cross-linking, and dissolution, samples were also taken from the final particle suspension (Figure 6 – sample B). To determine the bioburden, two samples were analyzed: one from the particle suspension and one from the supernatant after centrifuging the suspension (3000*g*, 3 min).

In addition to this standard protocol, test series were also carried out in which coprecipitated particles were dissolved with EDTA without prior cross-linking with glutaraldehyde. Therefore, the last sample here was taken after resuspension in EDTA solution (Figure 6 – sample A).

The respective samples were serially diluted (undiluted to 1:1000), 100 µL of each dilution was spread on agar plates and incubated for one day at 37 °C. Possibly growing colonies were counted.

## References

[1] Sen Gupta A (2019). Shock.

[2] Coll-Satue C, Bishnoi S, Chen J, Hosta-Rigau L (2021). Biomater Sci.

[3] Winslow R M (2006). Vox Sang.

[4] Bäumler H, Georgieva R (2010). Biomacromolecules.

[5] Zhang Y, Chan H F, Leong K W (2013). Adv Drug Delivery Rev.

[6] Prapan A, Suwannasom N, Kloypan C, Chaiwaree S, Steffen A, Xiong Y, Kao I, Pruß A, Georgieva R, Bäumler H (2019). Coatings.

[7] Suwannasom N, Smuda K, Kloypan C, Kaewprayoon W, Baisaeng N, Prapan A, Chaiwaree S, Georgieva R, Bäumler H (2019). Nanomaterials.

[8] Chaiwaree S, Prapan A, Suwannasom N, Laporte T, Neumann T, Pruß A, Georgieva R, Bäumler H (2020). Pharmaceutics.

[9] (2021). Resolution No. 17 - Recognition of the Bovine Spongiform Encephalopathy Risk Status of Members.

[10] (2021). Freiheit von Tierseuchen - KVG.

[11] Bäumler H, Xiong Y, Liu Z Z, Patzak A, Georgieva R (2014). Artif Organs.

[12] Baeumler H, Georgieva R, Bäumler H (2007). Micro-particles, blood-substitute and method for forming same.

[13] Kao I, Xiong Y, Steffen A, Smuda K, Zhao L, Georgieva R, Pruss A, Bäumler H (2018). Artif Organs.

[14] Mansouri A (1985). Am J Med Sci.

[15] White J C, Beaven G H (1954). J Clin Pathol.

[16] Watson S W, Novitsky T J, Quinby H L, Valois F W (1977). Appl Environ Microbiol.

[17] Caroff M, Novikov A (2020). OCL: Oilseeds Fats, Crops Lipids.

[18] Schwarz H, Schmittner M, Duschl A, Horejs-Hoeck J (2014). PLoS One.

[19] Fung F M, Su M, Feng H-t, Li S F Y (2017). Sci Rep.

[20] Buehler P W, Boykins R A, Jia Y, Norris S, Freedberg D I, Alayash A I (2005). Anal Chem (Washington, DC, U S).

[21] Habeeb A F S A, Hiramoto R (1968). Arch Biochem Biophys.

[22] Matei A, Puscas C, Patrascu I, Lehene M, Ziebro J, Scurtu F, Baia M, Porumb D, Totos R, Silaghi-Dumitrescu R (2020). Int J Mol Sci.

[23] Ballantyne B, Jordan S L (2001). J Appl Toxicol.

[24] Gorman S P, Scott E M, Russell A D (1980). J Appl Bacteriol.

[25] Sabatini D D, Bensch K, Barrnett R J (1963). J Cell Biol.

[26] Russell A D, Hopwood D, Ellis G P, West G B (1976). The Biological Uses and Importance of Glutaraldehyde. Progress in Medicinal Chemistry.

[27] Banfi G, Salvagno G L, Lippi G (2007). Clin Chem Lab Med.

[28] Leive L (1965). Proc Natl Acad Sci U S A.

[29] Haque H, Russell A D (1974). Antimicrob Agents Chemother.

[30] Root J L, McIntyre O R, Jacobs N J, Daghlian C P (1988). Antimicrob Agents Chemother.

[31] Gangan M S, Athale C A (2017). R Soc Open Sci.

[32] Otto M (2009). Nat Rev Microbiol.

[33] Miragaia M, Thomas J C, Couto I, Enright M C, de Lencastre H (2007). J Bacteriol.

[34] (2015). Supragingival Microbes. Atlas of Oral Microbiology.

[35] Ramírez‐Arcos S, Goldman M, Murphy M F, Roberts D J, Yazer M H (2017). Bacterial Contamination. Practical transfusion medicine.

[36] Levy J H, Neal M D, Herman J H (2018). Crit Care.

[37] Stramer S L, Dodd R Y, Hoffman R, Heslop H, Weitz J I (2018). Transfusion-Transmitted Diseases. Hematology: Basic principles and practice.

[38] Wilson-Nieuwenhuis J S T, Dempsey-Hibbert N, Liauw C M, Whitehead K A (2017). Colloids Surf, B.

[39] Perrotta P L, Snyder E L, Michelson A D (2011). Platelet Storage and Transfusion. Platelets.

[40] Gottenbos B, Grijpma D W, van der Mei H C, Feijen J, Busscher H J (2001). J Antimicrob Chemother.

[41] Maillard J-Y (2005). Ther Clin Risk Manage.

[42] Russell A D (1999). J Hosp Infect.

[43] McDonnell G, Russell A D (1999). Clin Microbiol Rev.

[44] McGucken P V, Woodside W (1973). J Appl Bacteriol.

[45] Maillard J-Y (2002). J Appl Microbiol.

[46] Munton T J, Russell A D (1972). J Appl Bacteriol.

[47] Hill S D, Berry C W, Seale N S, Kaga M (1991). Oral Surg, Oral Med, Oral Pathol.

[48] Davison W M, Pitts B, Stewart P S (2010). Antimicrob Agents Chemother.

[49] Sawant R B, Jathar S K, Rajadhyaksha S B, Kadam P T (2007). Asian J Transfus Sci.

[50] Sowemimo-Coker S O (2002). Transfus Med Rev.

[51] Xiong Y, Steffen A, Andreas K, Müller S, Sternberg N, Georgieva R, Bäumler H (2012). Biomacromolecules.

[52] Walsh S E, Maillard J-Y, Russell A D, Catrenich C E, Charbonneau D L, Bartolo R G (2003). J Appl Microbiol.

[53] Umerska A, Strandh M, Cassisa V, Matougui N, Eveillard M, Saulnier P (2018). Biomolecules.

[54] Schnaitman C A (1971). J Bacteriol.

[55] Prachayasittikul V, Isarankura-Na-Ayudhya C, Tantimongcolwat T, Nantasenamat C, Galla H-J (2007). Acta Biochim Biophys Sin.

[56] Leive L (1965). Biochem Biophys Res Commun.

[57] Levy S B, Leive L (1968). Proc Natl Acad Sci U S A.

[58] Gray G W, Wilkinson S G (1965). J Gen Microbiol.

[59] Russell A D (1967). J Appl Bacteriol.

[60] Chew B P, Tjoelker L W, Tanaka T S (1985). J Dairy Sci.

[61] Hart J R (2000). Ethylenediaminetetraacetic Acid and Related Chelating Agents. Ullmann's Encyclopedia of Industrial Chemistry.

[62] Haney C R, Buehler P W, Gulati A (2000). Adv Drug Delivery Rev.

[63] Xiong Y, Liu Z Z, Georgieva R, Smuda K, Steffen A, Sendeski M, Voigt A, Patzak A, Bäumler H (2013). ACS Nano.

[64] Palmer A F, Sun G, Harris D R (2009). Biotechnol Prog.

[65] Xiong Y, Georgieva R, Steffen A, Smuda K, Bäumler H (2018). J Colloid Interface Sci.

[66] Kloypan C, Prapan A, Suwannasom N, Chaiwaree S, Kaewprayoon W, Steffen A, Xiong Y, Baisaeng N, Georgieva R, Bäumler H (2018). Artif Cells, Nanomed, Biotechnol.

